# Positive effects of robotic exoskeleton training of upper limb reaching movements after stroke

**DOI:** 10.1186/1743-0003-9-36

**Published:** 2012-06-09

**Authors:** Antonio Frisoli, Caterina Procopio, Carmelo Chisari, Ilaria Creatini, Luca Bonfiglio, Massimo Bergamasco, Bruno Rossi, Maria Chiara Carboncini

**Affiliations:** 1 PERCRO (Perceptual Robotics) Laboratory, Scuola Superiore Sant’Anna, Viale Rinaldo Piaggio, 34, 56025, Pontedera, Pisa, Italy; 2 Neurorehabilitation Unit, Department of Neurosciences, Azienda Ospedaliera Universitaria Pisana, Via Paradisa, 2, 56124, Pisa, Italy

**Keywords:** Upper extremity hemiparesis, Stroke, Robot-aided rehabilitation, Motor synergies

## Abstract

This study, conducted in a group of nine chronic patients with right-side hemiparesis after stroke, investigated the effects of a robotic-assisted rehabilitation training with an upper limb robotic exoskeleton for the restoration of motor function in spatial reaching movements. The robotic assisted rehabilitation training was administered for a period of 6 weeks including reaching and spatial antigravity movements. To assess the carry-over of the observed improvements in movement during training into improved function, a kinesiologic assessment of the effects of the training was performed by means of motion and dynamic electromyographic analysis of reaching movements performed before and after training. The same kinesiologic measurements were performed in a healthy control group of seven volunteers, to determine a benchmark for the experimental observations in the patients’ group. Moreover degree of functional impairment at the enrolment and discharge was measured by clinical evaluation with upper limb Fugl-Meyer Assessment scale (FMA, 0–66 points), Modified Ashworth scale (MA, 0–60 pts) and active ranges of motion. The robot aided training induced, independently by time of stroke, statistical significant improvements of kinesiologic (movement time, smoothness of motion) and clinical (4.6 ± 4.2 increase in FMA, 3.2 ± 2.1 decrease in MA) parameters, as a result of the increased active ranges of motion and improved co-contraction index for shoulder extension/flexion. Kinesiologic parameters correlated significantly with clinical assessment values, and their changes after the training were affected by the direction of motion (inward vs. outward movement) and position of target to be reached (ipsilateral, central and contralateral peripersonal space). These changes can be explained as a result of the motor recovery induced by the robotic training, in terms of regained ability to execute single joint movements and of improved interjoint coordination of elbow and shoulder joints.

## Background

Impairment of upper limb function is one of the most common sequelae following stroke; in particular arm function is found to be altered in 73% to 88% of first time stroke survivors (infarctions only), and 55% to 75% still experience problems that impair their activities of daily living for up to 3 to 6 months or more [1,2].

Impairments limit the patient’s autonomy in daily living and may lead to permanent disability [3]. The deficits are typically characterized by weakness of specific muscles [4], lack of mobility between structures at the shoulder girdle [5], incorrect timing of components within a movement pattern [6,7] and loss of interjoint coordination [8]. Consequently goal directed movements in hemiplegic patients are characterized by lower movement amplitude, prolonged movement time, segmented trajectories and abnormal pattern of muscle activation. Compensatory motor strategies, characterized by adaptations to muscle imbalance [9], are commonly adopted by stroke patients in attempt to overcome these impairments.

Various rehabilitation interventions to improve skill reacquisition have shown promising results in overcoming motor impairment after stroke [10].

High intensity and task specific upper limb treatment consisting of active, highly repetitive movement is one of the most effective approaches to arm function post-stroke restoration [11-13]. Recent studies moreover suggest that given appropriate training, motor improvements of the upper limb can continue well into the chronic stage of stroke [14-16].

The use of robot devices in rehabilitation can provide high intensity, repetitive, task specific and interactive treatment of the impaired upper limb and an objective, reliable mean of monitoring patients progress. Systematic review confirms the potential for robotic assisted devices to elicit improvements in upper limb function [17,18]. Moreover virtual reality provided a unique medium where therapy can be provided within a functional, purposeful and motivating context and can be readily graded and documented [19]. The cortical reorganization and associated functional motor recovery after virtual reality in patient with chronic stroke are documented also by fMRI [20].

While several studies have already investigated the effects of robot assisted training in planar movements performed in the horizontal plane [21], the effect of training on the control and production of multi-joint and spatial functional arm movements, including movements against gravity, in hemiparetic subjects has received less attention.

It has been already shown that stereotyped movement patterns [8] due to abnormal muscle co-activation result in a reduced active range of motion against gravity. In particular providing antigravity limb support, leads to a reduction of the abnormal coupling between shoulder abduction/elbow flexion [22] and promising results have been found in the robotic training of patients with antigravity vertical movements that involve shoulder elevation [23]. Moreover orthoses providing only passive gravity assistance to the arm in reaching movements can induce comparable clinical improvements to those obtained with robotic training [24].

In this study we have investigated the effects of robot aided training on the recovery of spatial reaching movements, with a focus on point-to-point reaching movements performed in different directions, analysing how muscle imbalance in stroke influences the process of motor recovery in terms of regain of smooth movement, interjoint coordination and agonistic/antagonistic muscle recruitment.

A robotic treatment was administered through the L-EXOS [25,26], a robotic exoskeleton for the upper limb, in a group of nine patients with chronic hemiparetic stroke. Exoskeleton robotic systems allow to execute full spatial multi-joint functional arm movements, including elevation movements with shoulder abduction, providing either variable gravity support or active assistance to the impaired arm [27].

To assess the carry-over of the observed improvements in movement during training into improved function, changes in movement execution and smoothness of motion were analysed through a kinesiologic assessment, consisting in the motion and dynamic electromyographic analysis of reaching movements performed before and after training.

The kinesiologic performance (movement time, smoothness of motion) was then analysed in relation to the changes in the EMG pattern of agonist–antagonist muscle co-activation and shoulder-elbow interjoint coordination.

## Methods

### Participants

Nine right hemiparetic subjects (aged 61.4 ± 14.1 years) participated to the study. They had sustained a single left stroke between 3 and 9 years previously, leading to right-side hemiparesis. All subjects were able to understand simple commands and to perform a reaching movement with the affected arm. They had no other neurological, neuromuscular or orthopaedic disorders and no visual deficit. Explicit exclusion criteria were perceptual, apraxic or major cognitive deficits, shoulder sub-luxation or pain in the upper limb, spasticity (single muscle Modified Ashworth Scale Score > 2). In addition, subjects were excluded if they had occipital, cerebellar or brainstem lesions. Clinical and demographic data with baseline clinical assessment at the enrolment are reported in Table 1.

**Table 1:** Demographic data and clinical scores for hemiparetic subjects

**Pt.**	**Sex**	**Age**	**Type of stroke**	**Site of stroke**	**Fugl-Meyer score (66)**	**Ashworth score (60)**
1	M	79	Haemorrhagic	posterior portion of the left lateral ventricle roof with an extension corresponding to the semi oval centre	36	19,5
2	M	72	Haemorrhagic	temporo-parietal, cortical- subcortical left side	52	11
3	M	68	Ischemic	parieto-occipital, cortical-subcortical left side	12	10,5
4	M	69	Ischemic	extensive lesion in the left parietal side	56	5
5	M	58	Haemorrhagic	intra-parenchymal lenticular-capsular left collection	57	4,5
6	F	42	Haemorrhagic	midbrain-thalamus left lesion	12	12
7	M	58	Haemorrhagic	temporo-parietal left side	43	7,5
8	M	37	Haemorrhagic	nucleo-capsule-radiata left side	37	8,5
9	M	70	Haemorrhagic	temporo-parietal left side	17	7,5

The study was approved by the local ethical committee; subjects were informed of the procedure and gave their informed consent to participate to the study.

Both prior to and after the robotic training, the subjects were tested clinically for upper limb motor function by the upper limb Fugl-Meyer (FMA) scale (0–66 points) [28], muscle spasticity by the Modified Ashworth (MA) scale rating stiffness in 15 different muscle groups of the upper limb (0–60 points) [29] and in terms of active Ranges of Motion. Ranges of motion, both active and passive, were measured by means of a long-arm goniometer with a 360° scale marked in one degree increments.

### Kinesiologic assessment

The subjects were also submitted, before and after robotic training, to a kinesiologic test of selected movements. Free arm reaching and grasp of an object positioned on a horizontal plane were recorded by means of an integrated motion capture system and surface EMG recording system (Elite-BTS) composed of an 8 channel electromyography system and 6 cameras, with 100 Hz acquisition frequency and a position accuracy of 1.5 mm in the adopted configuration.

Motion capture was made with 4 surface markers of 0.5 cm diameter, covered with reflective material; placed at four points (sternum-clavicle junction, shoulder, elbow, wrist).

EMG signals were acquired from four muscle groups, respectively the couple of agonists/antagonists involved in elbow flexion/extension, triceps brachii (TB) and biceps brachii (BB), and in shoulder extension/flexion, anterior deltoid (AD) and posterior deltoid (PD). The activity was obtained using standard Ag/AgCl bipolar surface electrodes (10 mm diameter). Electrodes were positioned over the border of the distal third of the muscle halfway between the innervations zone and the distal tendon parallel to the muscle fibres according to SENIAM guidelines [30].

**Figure 1:**
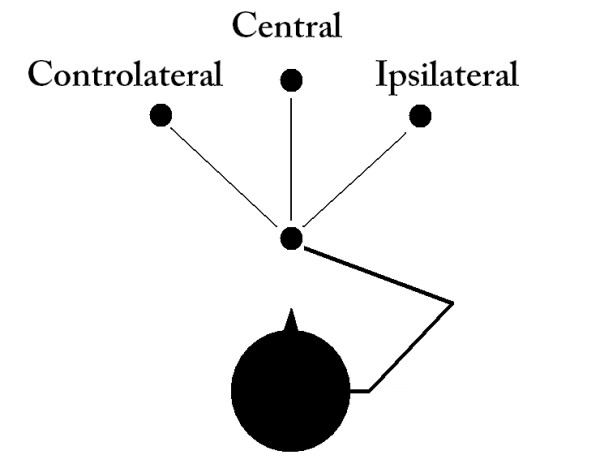
The patients’ movements were executed to reach different targets placed at ipsilateral, central and contralateral positions.

Each patient was asked to sit at a table on which a target to be reached was placed at about 30 cm distance from the subject; at the beginning patients had the right upper limb approximately 90 degrees flexed in the transverse plane, than they were invited to perform the extension of the arm (outward movement) to bring the limb to the target, to extend the palm to reach the target and, at the end, to bring back the limb at the initial position (inward movement). The patients were asked to operate at their own preferred speed to reach 3 different positions on the transverse plane (in contralateral, in central and in ipsilateral positions, according to [31]), as shown in Figure 1, and to repeat the same movement 3 times for each target position.

Seven volunteers (aged 47.14 ± 16.55 years), right-handed, were enrolled as well in a healthy control group. They were asked to perform the same kinesiologic procedures, to acquire a baseline of motion data to be used as a benchmark for the patients’ group.

### Robotic training

**Figure 2:**
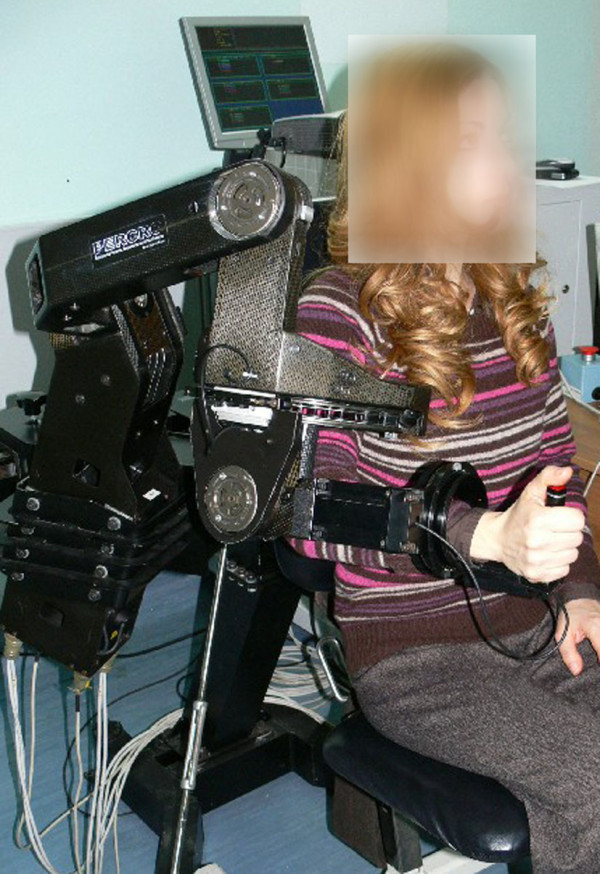
The L-Exos robotic exoskeleton.

The training session consisted of repetitive, goal directed forward reaching movements actively performed by the subject (partially assisted by the robotic device). In accordance to previous studies [21], robot aided therapy consisted of 3 one-hour rehabilitation sessions per week for a total of 6 weeks (i.e., 18 therapy sessions). Training was conducted by means of the L-Exos (see Figure 2, [25]), an active robotic exoskeleton, that can provide either active guidance during the execution of some exercise or gravity support for the weight arm. The exoskeleton has four actuated Degrees of Freedom (DoFs) with anthropomorphic kinematics, so that active assistance can be provided for shoulder abduction/adduction, flexion-extension, internal/external rotation and for elbow extension/flexion, and one passive DoF, corresponding to the wrist prono-supination.

**Figure 3:**
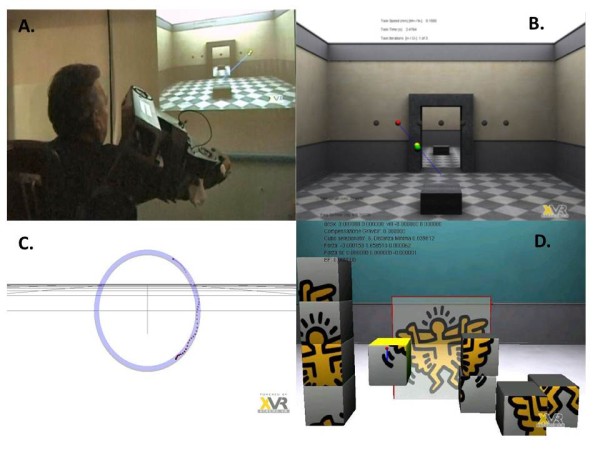
Panel A: one patient performing the robotic-aided therapy exercises in front of the VR scenario. Panels B,C,D: The virtual scenarios used for the training.

During the training sessions the patients were sat down on a seat, with their right forearm wearing the exoskeleton and a video projector displaying frontally the virtual scenario (see Figure 3, panel A).

Each rehabilitation session consisted of three different virtual reality mediated exercises [32].

In the first exercise, different fixed virtual targets were displayed to the patient as grey spheres disposed on a horizontal row, (Figure 3, panel B). When one of the fixed targets was activated, the patients were instructed to reach it, by actively following the position of a yellow sphere moving automatically towards the target with a motion generated along a straight trajectory according to a minimum jerk model [33]. The patients were actively aided by the robot, providing an impedance-based assistance proportional to the position error between the yellow sphere and patient hand’s position. Speed of the task was adjustable among three different values (5 cm/s, 10 cm/s and 15 cm/s), as well as the position of the targets to be reached, in terms of height and depth. Each patient performed training exercises at two different speeds. Speed of target, position of virtual targets (height, depth) were decided during a trial session at the enrolment, on the basis of the outcomes of the clinical evaluation, by adjusting the difficulty of the exercise to the capabilities of each patient.

In the second exercise (Figure 3, panel C) the patients were asked to draw a circular trajectory in a virtual plane, in the two conditions with and without the robot impedance-based assistance, providing a constraint along the requested trajectory. The task required an active interjoint coordination of elbow and shoulder joints to generate circular trajectories. In both conditions no active driving force was applied to the patients’ limb, but only support for gravity compensation until the patients were able to autonomously perform the task.

In the third exercise (Figure 3, Panel D) the patients were asked to assemble nine cubes in a virtual puzzle. Collisions with and between the cubes were simulated as computed contact forces applied at the patient’s hand by the robot. Two facilitation strategies were provided through the robot to perform the task: amplification of movement by an adjustable gain between the distance covered by the arm and the associated movement generated in the simulation, and active compensation of the arm’s weight, as in the second exercise. The levels of adjustable gain and active compensation were decided during a trial session at the enrolment according to the motor impairment of each patient, in order to make each patient able to autonomously complete the requested exercises.

### Data analysis

Kinesiologic individual data collected before and after training period in free arm motion were filtered with a smoothing low-pass filter to eliminate components above 200 Hz. Velocity was computed from position data with a central finite difference method over 22 subintervals.

Two main Performance Indexes (PI) were computed based on kinesiologic assessment of arm movement, analysing execution time (PI1) and smoothness (PI2) of the movement. The smoothness and regularity of the arm movement was measured by counting the number of local minima in the wrist velocity profile (PI2). Both indexes were then correlated to clinical scale assessment (FMA and MA scores).

Moreover the same indexes were recomputed on the outward and inward segments of the whole trajectory, obtained by automatic segmentation according to the following procedures.

The reaching movements, measured as displacement of the wrist, was segmented into three phases (outward movement, achievement of target, inward movement) on the basis of the displacement and speed profiles. The start of the outward phase was identified as the reference instant when a quarter of the maximum speed was achieved in each repetition, while the starting instant of the inward phase was identified at a quarter of the negative peak of the maximum negative speed.

**Figure 4:**
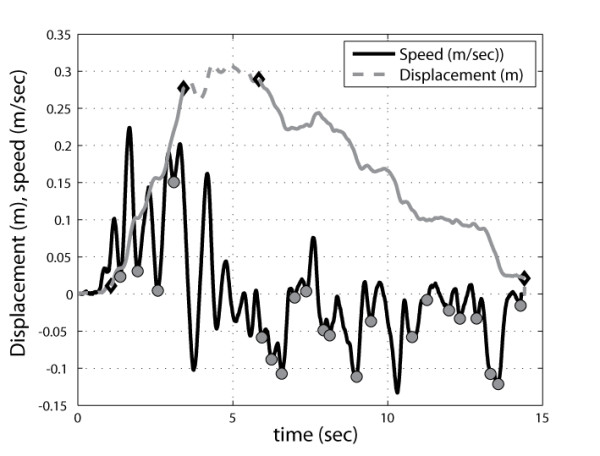
Example of automatic identification of kinematic features associated to the ulna displacement.

Then times of the outward (PI1a) and inward (PI1b) movements were computed, and number of minima in the velocity profiles associated to the same phases (PI2a, PI2b). Figure 4 shows an example of automatic extraction of duration of outward and inward phase (continuous line in grey), while on the same plot the count of local minima in the velocity profile is shown.

Joint angles variations for shoulder and elbow were computed respectively as the angle between the segments sternum-shoulder-elbow and shoulder-elbow-wrist.

Interjoint coordination was assessed by considering only the outward phase of the reaching movement, obtained by the same automatic segmentation algorithm presented above, and computing the diagrams of shoulder vs. elbow angle variations. For reaching tasks towards contralateral and central targets, since a coordinated rotation of shoulder and elbow joints acting in synergy is required, the interjoint coordination was evaluated computing the linear correlation coefficient between each pair of shoulder and elbow angles associated to movement execution. For reaching tasks towards ipsilateral targets, since the same movement can be executed with single joint movements of either shoulder or elbow joints, according to the motion strategy adopted by each subject, an index of coordination was computed as the ratio of maximum elbow (Δα_*ELB*_) to shoulder (Δα_*SH*_) excursion, according to the formula $\frac{\max\left(\Delta \alpha_{\mathrm{ELB}}\right)}{\max\left(\Delta \alpha_{\mathrm{SH}}\right)}$.

**Figure 5:**
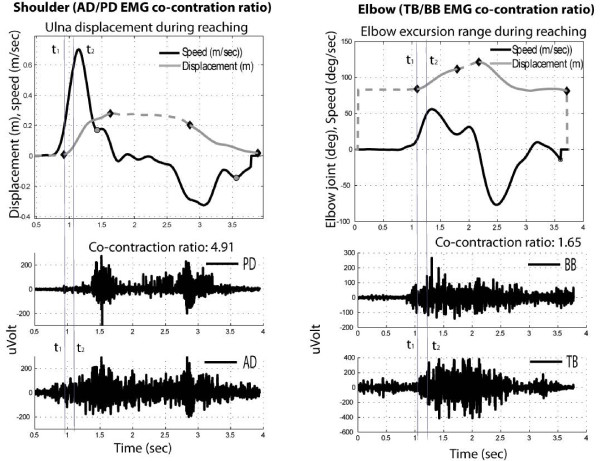
** Example of co-contraction ratio computation for AD/PD associated to ulna movement and for TB/BB associated to elbow angle variation (black line indicate velocity, grey line ulna displacement or elbow angle variation). Vertical grey lines indicate the time window used for the computation [t**_**1**_**-t**_**2**_**].**

All EMG signals were band-pass filtered (30–1.000 Hz). The co-contraction index(CCI) of activation of agonistic/antagonistic muscles for the couples TB/BB and AD/PD were then calculated to provide a normalized score evaluating co-contraction before and after the training period. The co-contraction index was computed only in the time window corresponding to the acceleration peak, ranging from the onset of movement (t_1_) as defined before up to half of the velocity peak (t_2_) (see Figure 5), as the ratio of the root mean square (RMS) of the EMG signals of agonist and antagonist muscles [34], according to the formula:

(1) $$\text{CCI}\left(\text{ag}/\text{ant}\right)=\frac{{\text{EMG}}_{ag,RMS}}{{\text{EMG}}_{ant,RMS}}=\frac{\sqrt{\int_{t_{1}}^{t_{2}}{\left[{\text{EMG}}_{\mathrm{ag}}\right]}^{2}dt}}{\sqrt{\int_{t_{1}}^{t_{2}}{\left[{\text{EMG}}_{\mathrm{ant}}\right]}^{2}dt}}$$

The kinematic reference used for the computation of the time window [t1-t2] was chosen as the ulna displacement for the CII1 of AD-PD muscles and as the elbow angle for the CII2 of TB/BB muscles, as shown in the example reported in Figure 5. In Figure 5, right side, it is possible to see how the antigravity action required to the biceps to sustain the weight of the forearm during all the movement, induces an associated co-activation of the triceps.

To make the scores more intuitively interpretable so that a high score reflected more normal function, the ratio of agonist/antagonist was used in preference to the ratio of antagonist/agonist, as by previous studies [34].

Statistical analysis was conducted with parametric tests. Two-sided *t*-test for paired samples was used to assess if changes in scores from admission to discharge were statistically significant in the patients’ group, linear regression analysis and Pearson correlation coefficients were used for the analysis of correlation among data. Post-hoc Tukey HSD test was used to compare the healthy control group with the patient’s group.

## Results

### Clinical and kinematic assessment

We observed a significant improvement in FMA from 35.8 ± 18.2 before training to 40.3 ± 17.6 after training (t(8) = 3.3, p = 0.006) and a reduction in MA scale from 9.6 ± 4.5 to 6.3 ± 5.0 (t(8) = 4.5, p = 0.001).

Significant improvements were found statistically associated to the execution of active movements. In particular we observed an increase of active range of motion for both shoulder and elbow joints, as reported in Table 2.

**Table 2:** Active range of motion changes after treatment (* p < 0.05, ** p < 0.01)

		**Admission (deg)**	**Discharge (deg)**	**p**
**Shoulder**	Abduction*	105 ± 61.4	112.2 ± 56.3	0.021
	Flexion	97.8 ± 76.7	108.3 ± 69.6	0.198
	Extension**	40 ± 22.8	52.8 ± 15.4	0.008
	Internal Rotation	56.7 ± 34.2	58.3 ± 35	0.438
	External Rotation**	54.4 ± 25.5	66.7 ± 21.4	0.008
**Elbow**	Flexion*	123.3 ± 21.2	130.6 ± 14.5	0.038
	Extension*	13.1 ± 12.1	6.7 ± 10	0.018

### Spatial-temporal assessment of movement

**Figure 6:**
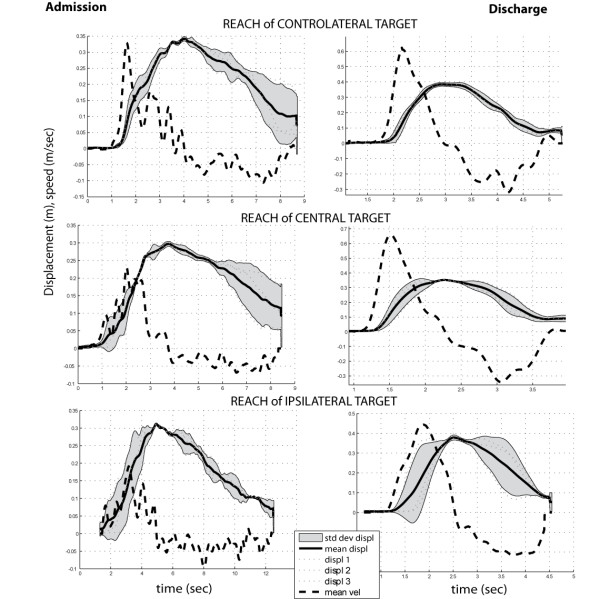
Example of ulna displacement profile averaged over three repetitions to reach a target for patient #1 (grey shaded area represent standard deviation, continuous and dashed line represent respectively mean ulna displacement and velocity averaged over three repetitions).

In Figure 6, the average profile of ulna with associated speed are reported for one patient before and after training, during the execution of 3 reaching movements. The characteristic double peak profile in speed is clearly visible, as well as the increase of smoothness of movement after robotic training. Local minima in the speed profile can account for the smoothness of movement.

Table 3 reports changes obtained in performance indexes PI1 and PI2 and in co-contraction ratio CII1 for AD/PD and CII2 for TB/BB. As a benchmark, the kinematic parameters observed in the healthy control group are reported in the last column of Table 3.

**Table 3:** Mean values ± standard deviation of performance indexes (* p < 0.05, ** p < 0.01). Reported statistics refer to the comparison between admission and discharge in the patients’ group

Performance Index		Contralateral movement			
		Admission	Discharge	p	Healthy Control
PI1	Total time (s)	**4.02 ±1.74**	**3.11 ±1.18**	0.012*	1.41 ± 0.72
PI2	Total smoothness	**4.19 ±3.17**	**2.33 ±2.76**	0.004**	0.19 ± 0.4
CCI1	AD-PD muscles	**1.64 ± 0.93**	**2.49 ± 1.94**	0.05*	7.17 ±2.99
CCI2	TB-BP muscles	1.50 ± 0.77	1.41 ± 1.02	0.91	0.45 ±0.27
Performance Index		Central movement			
		Admission	Discharge	p	Healthy Control
PI1	Total time (s)	**4.10 ±2.10**	**3.07 ±1.08**	0.007**	1.32 ± 0.72
PI2	Total smoothness	**5.15 ±4.76**	**2.74 ±2.30**	0.006**	0.24 ± 0.54
CCI1	AD-PD muscles	**2.24 ± 0.80**	**3.08 ± 2.21**	0.03*	7.9 ±2.92
CCI2	TB-BP muscles	1.39 ± 0.84	1.37 ± 0.87	0.84	0.41 ±0.29
Performance Index		Ipsilateral movement			
		Admission	Discharge	p	Healthy Control
PI1	Total time (s)	**5.25 ±2.84**	**3.15 ±1.14**	0.0004**	1.64 ± 1.04
PI2	Total smoothness	**7.00 ±6.69**	**2.63 ±2.56**	0.001**	0.62 ± 0.74
CCI1	AD-PD muscles	**1.24 ± 0.64**	**1.89 ± 1.41**	0.02*	4.87 ±2.75
CCI2	TB-BP muscles	1.31 ± 0.95	1.36 ± 1.22	0.81	0.46 ±0.32

**Figure 7:**
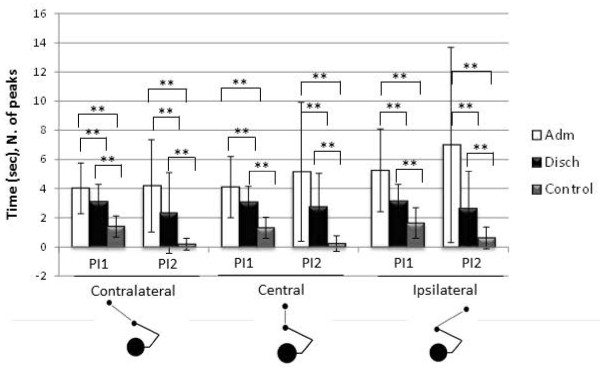
Graphical representation of kinesiologic indexes PI1 (total time) and PI2 (smoothness) changes before and after training (* p < 0.05, ** p < 0.01).

Both performance time and smoothness decreased in all conditions, with higher changes after training for ipsilateral and central targets than contralateral ones, as it is clearly visible in the graphical representation in Figure 7. Comparable performance values were reached for PI1 and PI2 at discharge in all target conditions.

**Figure 8:**
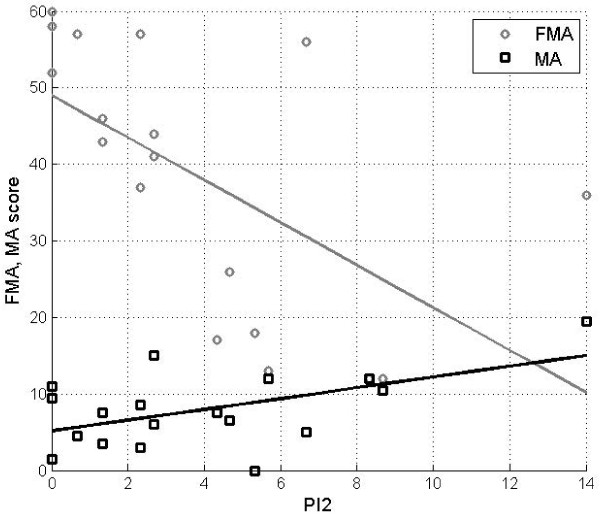
Correlation of FMA and MA score with index PI2 (smoothness index to reach a central target).

Both parameters PI1 and PI2 were found to be significantly correlated with clinical assessment scores obtained with FMA and MA scales. In Table 4 we report the correlation values for PI1 and PI2 computed in the reaching movement towards a central target. In particular the fluctuations in velocity profile (PI2) during reaching presented the strongest correlation with clinical assessment, as graphically reported in Figure 8.

**Table 4:** Pearson correlation coefficients for correlation of PI1-2 (central target) vs. FMA and MA clinical scores (* p < 0.05, ** p < 0.01)

		**PI1**	**PI2**
FMA	R	−.501^*^	−.589^*^
	P	**.034**	**.010**
MA	R	.427	.530^*^
	P	.077	**.0024**

**Figure 9:**
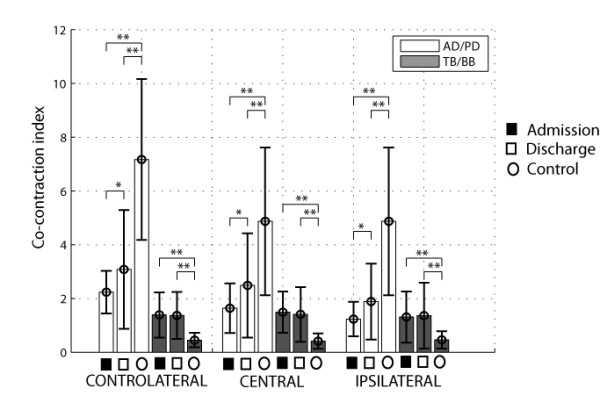
Co-contraction ratio for different couples of agonist/antagonist muscle, pre and after training (* p < 0.05).

Considering the recruitment of muscle groups in the execution of movement, as shown in Figure 9, only significant changes of the co-contraction index CCI1 (AD/PD) associated to movement of shoulder extension/flexion were found, in all conditions, while no significant difference was found for the co- contraction index CII2 (TB/BB) associated to elbow flexion/extension. No significant correlation was observed of the co-contraction indexes with clinical assessment scores from FMA and MA.

This is coherent with the proposed exercises requiring limited involvement of elbow joint, and a strong involvement of shoulder flexion/extension to move the hand forward and backward. The respective baseline measured in the healthy control group for the co-contraction index is reported as well in Figure 9. It is possible to see that there is a statistically significant difference among the two groups (patients vs. healthy control groups), and in particular that the CCI1 (AD/PD) is higher in the control group. For the CII2 (TB/BB) the prevalent action of the antagonist (BB) during the outward phase observed in the control group is due to the antigravity action required to sustain the forearm, that leads to an associated co-activation of the triceps in the patients’ group.

### Outward vs. inward movement

The analysis of movement time and smoothness on the segmented inward and outward phases is reported in Table 5. It is possible to notice how a statistically significant change in performance is always reached for ipsilateral targets, while for central targets performance is unchanged for inward time and smoothness, and for contralateral targets only the inward smoothness is improved at discharge.

**Table 5:** Comparison of outward vs. inward movement performance

**Performance Index**		**Contralateral movement**			**Central movement**			**Ipsilateral movement**		
		**Admission**	**Discharge**	**p**	**Admission**	**Discharge**	**p**	**Admission**	**Discharge**	**p**
PI1 a	Outward time (s)	1.30 ±0.57	1.10 ±0.60	0.172	**1.26 ±0.69**	**0.93 ±0.35**	0.015*	**1.38 ±0.71**	**0.99 ±0.54**	0.008**
PI1 b	Inward time (s)	1.40 ±1.15	1.13 ±0.52	0.124	1.33 ±0.72	1.22 ±0.57	0.483	**2.07 ±1.84**	**1.09 ±0.27**	0.014*
PI2 a	Outward smoothness	1.22 ±1.31	0.81 ±1.64	0.170	**1.19 ±1.36**	**0.59 ±0.69**	0.030*	**1.44 ±1.40**	**0.56 ±1.01**	0.003**
PI2 b	Inward smoothness	**1.33 ±1.80**	**0.67 ±0.78**	0.034*	1.48 ±1.70	1.22 ±1.31	0.483	**2.70 ±3.94**	**0.85 ±0.91**	0.030*

### Interjoint Coordination

**Figure 10:**
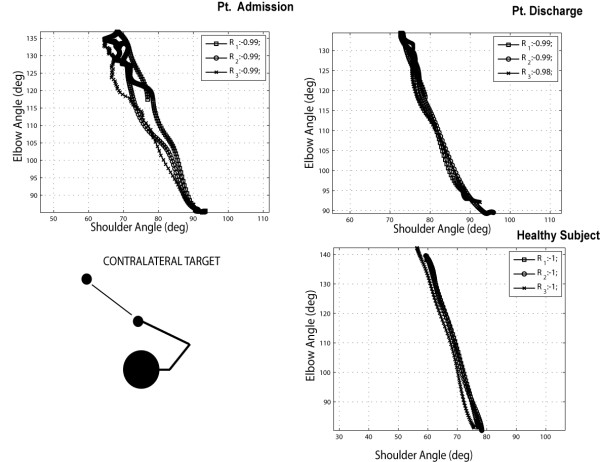
** Interjoint coordination between elbow and shoulder angles during 3 repetitions of an outward reaching movement towards a contralateral target (Pt. 7 vs. healthy volunteer). Reported R**_**i**_**values in the legend indicate the observed elbow-shoulder angle correlation values.**

The diagram in Figure 10 shows elbow vs. shoulder angles during the outward phase of movement of one patient (n. 7) compared with the same movement performed by one healthy subject of the control group during three repetitions of reaching a target in the contralateral space.

**Figure 11:**
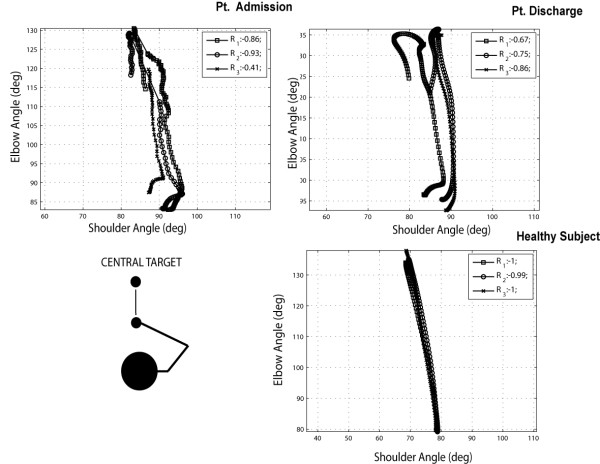
Interjoint coordination between elbow and shoulder angles during 3 repetitions of an outward reaching movement towards a central target (Pt. 7 vs. healthy volunteer).

From the diagram, it appears that the movement execution in terms of shoulder-elbow angles, is not significantly affected by the training and different from the execution of the healthy subject. It is interesting to compare the performance in the case of the reach of a central target (Figure 11). Again in the healthy volunteer we can notice a strong correlation between shoulder-elbow angles, while the patient presents a more segmented diagram with lower correlation values.

**Figure 12:**
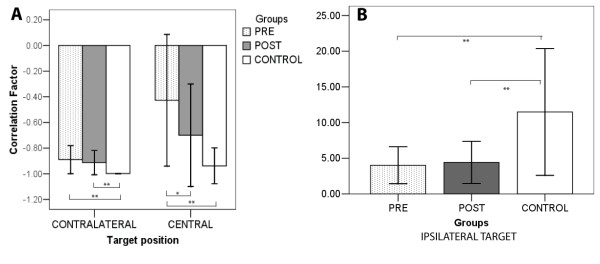
Interjoint correlation coefficients before and after training according to target location for central and contralateral targets.

The difference in interjoint coordination between the executions of contralateral and central reaching, measured in terms of correlation coefficients, is graphically reported in Figure 12, panel A, for all patients and for the healthy control group.

In the reach of a contralateral target the correlation coefficient is almost equal to −1 (−1.00 ± 0.00) in the control group, and appears to be well preserved in the patient’s group both before (−0.89 ± 0.11) and after (−0.91 ± 0.09) training with no statistically significant effect of the training (p = 0.27). In the case of reach to a central target, the movement executed in the healthy control group presents a high correlation coefficient (−0.94 ± 0.14) between elbow and shoulder angles, while in the patient’s group we observe a significant increase of the correlation coefficient, from −0.42 ±0.51 to −0.69 ± 0.39 (p < 0.0004).

**Figure 13:**
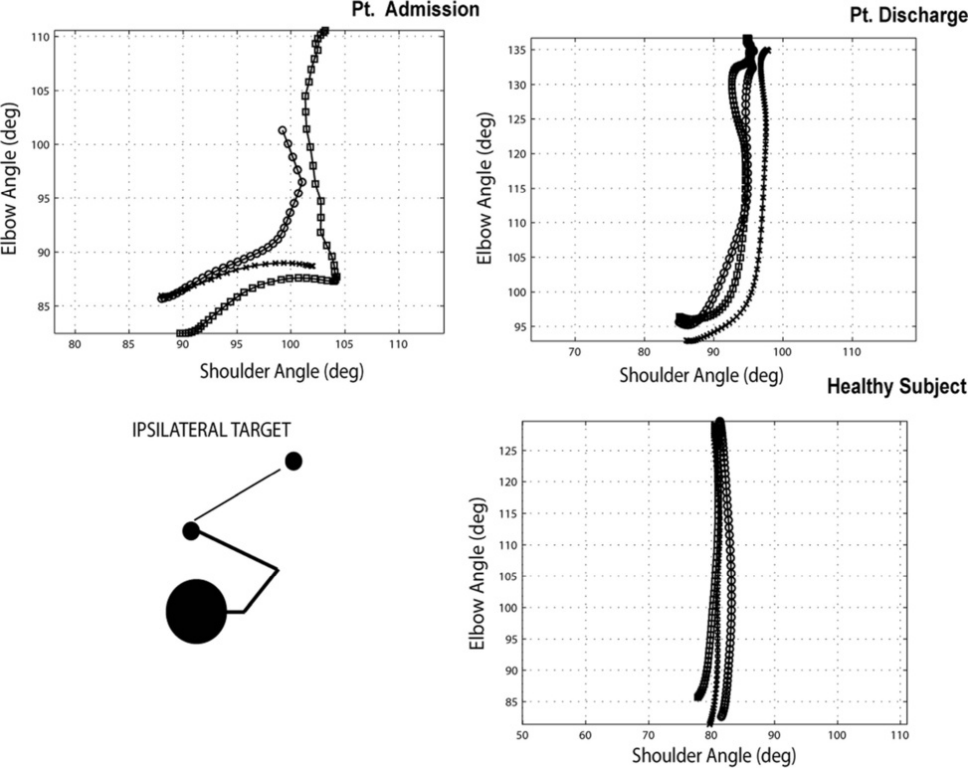
Interjoint coordination between elbow and shoulder angles during 3 repetitions of an outward reaching movement towards a ipsilateral target (Pt. 7 vs. healthy volunteer).

The analysis of the same movement executed towards an ipsilateral target cannot be performed in terms of correlation between shoulder and elbow angles. This is because, as it is visible in Figure 13, the movement is performed mainly with single joint movements of shoulder and elbow joints. The main qualitative difference that we notice in the performance after stroke is the reduced involvement of the elbow joint in movement execution.

The effects of training can be measured in terms of maximum elbow (Δα_*ELB*_) to shoulder (Δα_*SH*_) excursion. As reported in Figure 12, panel B, we did not observe any change of this index before (4.01 ± 2.59) and after training (4.4 ± 2.96), while higher values were attained in the healthy control group (11.48 ± 8.89), denoting a different motion strategy with higher recruitment of elbow joint in reaching directed to ipsilateral targets.

## Discussion

The recovery of motor capabilities during the chronic phase of impairment after stroke with a rehabilitation treatment is documented in literature [14,15,35].

Within the group of patients that participated to this study, we observed coherently with previous research an increase in the FMA and reduction in the MA scores, the former corresponding to an improvement of the motor performance, the latter in a reduction of the muscular spasticity.

In addition, the instrumental study of the reaching performance showed that robotic training produced positive effects in movement execution, in terms of decreased execution time, improved movement smoothness and increased active joint ranges of motion.

Studies of development and recovery from neurological injury strongly suggest that smoothness is a result of learned, coordinative process, rather than a natural consequence of the structure of neuromuscular system [36]. The observed reduction of the movement irregularity indicates a better motor control, i.e. a more appropriate recruitment of agonists and antagonists. Abnormal spatial and temporal patterns of agonist- antagonist muscle activation and inadequate or maladaptive muscle co-activation have been reported in hemiparetic subjects. Abnormal co-activation has been related to diminished agonist motor unit recruitment, impaired antagonist inhibition or both [37] or to a decrease in the number of possible coordinative muscle synergies [38]. Several sources can explain muscle weakness observed after stroke: failure of voluntary motor neuron activation [39], loss of functioning motor units, changes in the properties of the remaining units, inappropriate spatial and temporal patterns of muscle activation, muscle fibre atrophy and contracture.

In our study, the observed functional changes were found to be associated to an improvement in the co- contraction index of proximal joints, in particular for shoulder extension and flexion (CII1 AD/PD), while no changes were observed in the co-contraction ratio of distal joints, i.e. elbow (CII2 TB/BB). From the comparison with data collected in the healthy group, it is evident that a co-activation of biceps and triceps muscle groups persists in the patient group, mainly due to the biceps activation to contrast the weight of the forearm during movement execution. On the contrary, the improved CII1 appears to be associated to the specific performed training exercises.

The decrease in motion range after stroke may be also a direct consequence of an enlarged antagonist muscle activity, due to the loss of ability to relax muscles not involved in the voluntary effort[40], accompanied by an increased resistance to movement [41]. This may be due to a decrease in the stretch- reflex threshold [31,42] and to limitations in its central regulation [43,44].

Additionally, there is evidence that the segmented nature of stroke patients’ arm movement can be attributed to a deficit in interjoint coordination [8]. In our group of patients, we observed an increase of interjoint coordination of shoulder and elbow joints for reaching movements directed towards a central target and differences in the changes of functional performance, dependent of the spatial position of the target to be reached, i.e. contralateral vs. ipsilateral targets. These experimental evidences might be explained taking into account the redundancy of the muscoskeletal system. For instance some movements, such as reaching an ipsilateral target, can be executed by choosing among different available motor strategies, selecting appropriate interjoint coordination and desired trajectory [45].

Hemiparetic patients normally exhibit abnormal joint coupling between shoulder and elbow joints [46] and loss of independent joint control. According to some theories [45,47,48], even if the issue is still controversial, recovery of movements from stroke in the upper extremity begins with the development of a flexor synergy (shoulder abduction-elbow flexion) followed by an extensor synergy (shoulder adduction- elbow extension) pattern [49], while isolated joint movements remain still compromised. This may result into the disruption of movement in terms of the required interjoint coordination.

The kinesiologic data from our study (Table 3) show that upper limb inward movements, usually performed within the “flexion synergy”, were more easily preserved in patients and less affected by the robotic training, if compared to outward movements. But the same finding can also be explained in terms of the higher involvement of shoulder extensor muscles in the outward phase, required for the antigravity support of the arm.

Similarly, despite the overall improvement of the kinesiologic parameters in the different conditions of free limb reaching task, we observed that the movement time (PI1a) and the smoothness (PI2a) of the outward movement into the contralateral space did not improve with training as much as in the ipsilateral space.

This aspect might be explained by analysing the main features of the performed movements in terms of interjoint coordination. It seems that the coordination pattern of shoulder and elbow joints is better preserved for reaching movements executed in the contralateral space, where we did not observe changes in the correlation factor between shoulder and elbow angles. This can be explained in terms of the “extensor synergy” required for, with a combined involvement of shoulder adduction and elbow extension [45].

It has been shown that the gravity compensation reducing the involuntary coupling between shoulder abduction and elbow flexion (flexor synergy) results then in a larger elbow extension during planar reach tasks [50,51]. In our group of patients, we did not observe neither change of the co-contraction index associated to elbow function (CII2 BB/TB) nor changes in the elbow to shoulder angles ratio in the execution of movement to ipsilateral targets. The training was mostly effective in terms of movement execution involving shoulder flexion/extension, even if single joint improvements were observed also in terms of active ranges of motion for shoulder abduction and elbow flexion/extension.

The study of kinematic changes is needed to better understand the observed improvements in motor performance [52,53], because the measurements at a functional level cannot differentiate between improvements at the motor recovery level or due to alternative compensatory strategies.

In this study we observed that the rehabilitation training, conducted on multi-joint and spatial reaching movements, can lead to significant improvements in the kinesiologic indexes related to the performance execution and motion smoothness, indicating a carry-over of the observed improvements during training into improved function.

Moreover the observed correlation of some kinesiologic parameters, such as the index of smoothness, with the clinical assessment, supports the development in robotic assisted rehabilitation of new methods for automatic assessment of patient’s performance, conducted continuously all over the training.

These changes were reflected also in a better interjoint coordination of shoulder and elbow joints, that probably contributes to the overall improvement of quality of movement, and better ability to execute single joint and out of synergy movements.

The analysis of kinesiologic performance in relation to the changes in the EMG pattern of agonist- antagonist muscle co-activation showed an improvement of the co-contraction index for shoulder flexion- extension, indicating a better expression of selective activation of agonist and antagonist muscles, and underlining the possible presence of plastic phenomena even in a long time (some years) from the event.

This study points out the importance of conducting the rehabilitation training of upper-limb after stroke with multi-joint and 3D spatial movements. In future works, the role of interjoint coordination and muscle recruitment in the recovery of movement from stroke and in the regained ability to explore different portions of the peri-personal space needs to be further investigated.

## Competing interest

The authors declare that they have no competing interests.

## Authors’ contribution

AF designed the study, managed the robotic and VR system design and implementation, supervised the robotic training, conducted the data analysis and interpretation of results, drafted the manuscript. CP participated to the robotic training with patients and to the clinical evaluation, contributed to the ideation of training exercises and to the organization of the training sessions. CC contributed to the medical supervision of the robotic training and to the recruitment and kinesiologic evaluation of the control group. IC was in charge of the clinical assessment of patients and conduction of kinesiologic tests. LB contributed to the data analysis and interpretation of results. MB contributed to the robotic and VR system design and implementation. BR was responsible for the clinical protocol, and supervised the clinical trial. MCC designed the study, was responsible for patients’ enrollment, conduction of clinical trial and medical supervision of the robotic training, contributed to the data analysis and drafted the manuscript. All authors read and approved the final manuscript.
